# How BRCA and homologous recombination deficiency change therapeutic strategies in ovarian cancer: a review of literature

**DOI:** 10.3389/fonc.2024.1335196

**Published:** 2024-03-08

**Authors:** Martina Arcieri, Veronica Tius, Claudia Andreetta, Stefano Restaino, Anna Biasioli, Elena Poletto, Giuseppe Damante, Alfredo Ercoli, Lorenza Driul, Anna Fagotti, Domenica Lorusso, Giovanni Scambia, Giuseppe Vizzielli

**Affiliations:** ^1^ Clinic of Obstetrics and Gynecology, “S. Maria della Misericordia” University Hospital, Azienda Sanitaria Universitaria Friuli Centrale (ASUFC), Udine, Italy; ^2^ Department of Biomedical, Dental, Morphological and Functional Imaging Science, University of Messina, Messina, Italy; ^3^ Medical Area Department (DAME), in Department of Medicine (DMED), University of Udine, Udine, Italy; ^4^ Department of Medical Oncology, “S. Maria della Misericordia” University Hospital, Azienda Sanitaria Universitaria Friuli Centrale (ASUFC), Udine, Italy; ^5^ Medical Genetics Institute, “S. Maria della Misericordia” University Hospital, Azienda Sanitaria Universitaria Friuli Centrale (ASUFC), Udine, Italy; ^6^ Department of Human Pathology in Adult and Childhood “G. Barresi”, Unit of Gynecology and Obstetrics, University of Messina, Messina, Italy; ^7^ Dipartimento per le Scienze Della Salute Della Donna, del Bambino e di Sanità Pubblica, UOC Ginecologia Oncologica, in Division of Gynecologic Oncology, Fondazione Policlinico Universitario Agostino Gemelli IRCCS, Rome, Italy

**Keywords:** ovarian cancer, homologous recombination deficiency, BRCA protein, BRCAness, HRD testing, PARP inhibitors, overall survival

## Abstract

About 50% of High Grade Serous Ovarian Cancer exhibit a high degree of genomic instability due to mutation of genes involved in Homologous Recombination (HRD) and such defect accounts for synthetic lethality mechanism of PARP inhibitors (PARP-i). Several clinical trials have shown how BRCA and HRD mutational status profoundly affect first line chemotherapy as well as response to maintenance therapy with PARP-i, hence Progression Free Survival and Overall Survival. Consequently, there is urgent need for the development of increasingly reliable HRD tests, overcoming present limitations, as they play a key role in the diagnostic and therapeutic process as well as have a prognostic and predictive value. In this review we offer an overview of the state of the art regarding the actual knowledge about BRCA and HRD mutational status, the rationale of PARPi use and HRD testing (current and in development assays) and their implications in clinical practice and in the treatment decision process, in order to optimize and choose the best tailored therapy in patients with ovarian cancer.

## 1 Introduction

Epitelian Ovarian cancer (EOC) represents the most lethal gynecological cancer with 314 000 women diagnosed with EOC and 207 000 deaths in 2020, according to the latest estimates (1). It consists of several histopathological entities, that can be basically reassumed into two categories: ovarian cancer type I includes low grade serous, mucinous, endometrioid, clear cell and transitional cell carcinomas which are suggested to be indolent, generally confined to the ovary and genetically stable; ovarian cancer type II includes high grade serous carcinoma (HGSOC), undifferentiated carcinoma and carcinosarcoma which appear to be more clinically aggressive and show high genomic instability (2, 3). Therefore, ovarian cancers subtypes differ not only for histopathological features but also for intrinsic and peculiar molecular and genetic pathways that influence carcinogenesis, growth and dissemination pattern of disease, response to chemotherapy and prognosis. High Grade Serous Carcinoma is the most common histological subtype of EOC; it has an aggressive behavior and is diagnosed mostly at advanced stages. It takes origin *de novo* from the tubo-ovarian surface epithelium showing as a probable precursor the serous tubal intraepithelial carcinoma (STIC), which is a dysplastic lesion within the tubal epithelium that have a proliferative p53 signatures (4). From a genetic point of view, it is widely known that it has a peculiar genetic profile characterized by a certain degree of genetic rearrangement and genomic instability due to the presence of different mutations (5). About 50% of HGSOC are estimated to have a defect in Homologous Recombination (HR), that is, along with NonHomologous End Joining Recombination (NHEJ), the main cellular pathway of double-stranded DNA damage repair. These mechanisms of repair show several differences, in example NHEJ is an error-prone process that fuses the two broken ends and functions throughout all cell cycle phases (except the M phase) whereas HR is an error-free pathway activated in the S/G2 phase that uses a genetically identical sister chromatid as a template for repair (6). NHEJ involves a multitude of proteins (like the most recent discovered one MRI/CYREN) and HR relies mainly on BRCA1-2 proteins, that are essential for the creation of the repair complex. In 15% of cases of HGSOC there is a germline mutation of the BRCA1/2 gene, in 6% of cases a somatic mutation of the BRCA1/2 gene and in 20% of cases a mutation of other genes involved in Homologous Recombination- for example, mutations and/or epigenetic silencing of the genes TR, ATM, RAD51/54, CHK1/2, NBS1, PALB2, PTEN and which equally determine a profile defined as “BRCA-ness “ (7). These mutations cause the cell to lose the ability to repair DNA damage caused by external insults, specifically those to the double helix, resulting in a condition defined as Homologous Recombination Deficiency (HRD). This occurrence favors tumor initiation, growth and evolution. With the acquisition of such molecular, genetic and biological knowledge, in the last decade a class of drugs has been identified. These drugs exploit HRD profile of tumor cells and at the same time interfering with other DNA repair mechanisms, determine the death of tumor cells. These are the polyADP - ribose polymers inhibitors (PARPi) represented by Olaparib, Niraparib and Rucaparib – approved, marketed and currently used in clinical practice. Their use as maintenance therapy after response to platinum-based first-line chemotherapy has led to a significant prolongation of the Progression Free Survival (PFS) and of overall survival (OS), particularly in BRCA-mutated patients. In the course of the years and studies, it has emerged that this effect is not limited to BRCA mutated patients but is also extended, albeit to a respectively decreasing extent, to BRCA-wild type patients yet with other mutations of the HR, to patients who do not have defects of HR also known as Homologous recombination Proficient (HRP) as well as patients with unknown mutational status (8). Bearing in mind that ovarian cancer tends to recur in 70% of cases within 3 years of first line chemotherapy (9), it is important to underline the role of maintenance therapy in delay disease progression, prevent recurrence and prolong the disease-free interval. The clinical application of this class of drugs is also extended to patients with relapsed platinum-sensitive ovarian cancer, in which cases PARPi have proven effectiveness in improving PFS in both BRCA mutant and wild-type patients (10).

To date, both the European and the American main oncological guidelines (11, 12) recommend the execution of the BRCA test in all patients with a new diagnosis of high-grade ovarian cancer, to search for somatic and germline mutations of BRCA1/2, and the execution of the HRD test, for the evaluation of the degree of genomic instability of the tumor, so as to identify the patients who would obtain the greatest benefit from PARP-inhibitor therapy, in the context of an increasingly customized and individualized precision medicine.

The purpose of this manuscript is to give and overview of the state of the art regarding the actual knowledge about BRCA mutational status and HRD and to analyze how this information is changing therapeutic strategies in HGSOC. **Table 1** summarizes the main studies included in the literature review.

**Table 1:** Main studies of literature review.

Title	Author	Year of publication
Cytoreductive surgery for advanced epithelial ovarian cancer in the poly(ADP-ribose) polymerase inhibitors era-Is it time for a new paradigm shift? A systematic review and meta-analysis	Peters ITA. et al. (13)	1990
Integrated genomic analyses of ovarian carcinoma	The Cancer Genome Atlas Research Network (5)	2011
Association of BRCA1 and BRCA2 Mutations With Survival, Chemotherapy Sensitivity, and Gene Mutator Phenotype in Patients With Ovarian Cancer	Yang D. et al. (14)	2011
RING domain–deficient BRCA1 promotes PARP inhibitor and platinum resistance	Wang Y. et al. (15)	2016
Methylation of all BRCA1 copies predicts response to the PARP inhibitor rucaparib in ovarian carcinoma	Kondrashova O. et al. (16)	2018
Parp inhibitors as maintenance treatment in platinum sensitive recurrent ovarian cancer: An updated meta-analysis of randomized clinical trials according to BRCA mutational status	Tomao F. et al. (10)	2019
ESMO–ESGO consensus conference recommendations on ovarian cancer: pathology and molecular biology, early and advanced stages, borderline tumours and recurrent disease.	Colombo N. et al. (11)	2019
BRCA Reversion Mutations in Circulating Tumor DNA Predict Primary and Acquired Resistance to the PARP Inhibitor Rucaparib in High-Grade Ovarian Carcinoma	Link KK. et al. (17)	2019
Niraparib in Patients with Newly Diagnosed Advanced Ovarian Cancer	González-Martín A. et al. (18)	2019
Olaparib plus Bevacizumab as First-Line Maintenance in Ovarian Cancer	Ray-Coquard I. et al. (19)	2019
PARP inhibitors in ovarian cancer: Sensitivity prediction and resistance mechanisms	Jiang X. et al. (20)	2019
PARP inhibitor resistance: the underlying mechanisms and clinical implication	Li H. et al. (21)	2020
Ovarian Cancer, Version 2.2020, NCCN Clinical Practice Guidelines in Oncology	Armstrong DK. et al. (12)	2021
Patient-derived organoids and high grade serous ovarian cancer: from disease modeling to personalized medicine	Nero C. et al. (22)	2021
Homologous recombination deficiency testing in advanced ovarian cancer: description of the ENGOT HRD European initiative	Pujade-Lauraine E. et al. (23)	2021
Molecular and clinical determinants of response and resistance to rucaparib for recurrent ovarian cancer treatment in ARIEL2 (Parts 1 and 2)	Swisher EM. et al. (24)	2021
BRCA1 Versus BRCA2 and PARP Inhibitors Efficacy in Solid Tumors:A Meta-Analysis of Randomized Controlled Trials	Li S. et al. (25)	2021
Applications of Proteomics in Ovarian Cancer: Dawn of a New Era	Ghose A. et al. (26)	2022
Fumagalli C, Betella I, Ranghiero A, Guerini-Rocco E, Bonaldo G, Rappa A, et al. In-house testing for homologous recombination repair deficiency (HRD) testing in ovarian carcinoma: a feasibility study comparing AmoyDx HRD Focus panel with Myriad myChoiceCDx assay	Fumagalli C. et al. (27)	2022
Alternative academic approaches for testing homologous recombination deficiency in ovarian cancer in the MITO16A/MaNGO-OV2 trial	Capoluongo ED. et al. (28)	2022
A Randomized, Phase III Trial to Evaluate Rucaparib Monotherapy as Maintenance Treatment in Patients With Newly Diagnosed Ovarian Cancer (ATHENA–MONO/GOG-3020/ENGOT-ov45)	Monk BJ. et al. (29)	2022
Efficacy of maintenance olaparib plus bevacizumab according to clinical risk in patients with newly diagnosed, advanced ovarian cancer in the phase III PAOLA-1/ENGOT-ov25 trial.	Harter et al. (30)	2022
Recent Insights into PARP and Immuno-Checkpoint Inhibitors in Epithelial Ovarian Cancer	Revythis A. et al. (31)	2022
European experts consensus: BRCA/homologous recombination deficiency testing in first-line ovarian cancer	Vergote I. et al. (32)	2022
PARP inhibitor resistance: the underlying mechanisms and clinical implication	Guffanti F. et al. (33)	2022
Efficacy of niraparib maintenance therapy in patients with newly diagnosed advanced ovarian cancer in phase 3 PRIME study: A subgroup analysis by response to first-line platinum-based chemotherapy	Yin R. et al. (36)	2022
Overall Survival With Maintenance Olaparib at a 7-Year Follow-Up in Patients With Newly Diagnosed Advanced Ovarian Cancer and a BRCA Mutation: The SOLO1/GOG 3004 Trial	DiSilvestro P. et al. (34)	2023
Efficacy and safety of PARP inhibitors for maintenance treatment of ovarian cancer, regardless of BRCA or HRD status: a comprehensive updated meta-analysis	Li Y. et al. (8)	2023
Association of location of BRCA1 and BRCA2 mutations with benefit from olaparib and bevacizumab maintenance in high-grade ovarian cancer: phase III PAOLA-1/ENGOT-ov25 trial subgroup exploratory analysis	Labidi-Galy SI. et al. (35)	2023
Olaparib plus bevacizumab first-line maintenance in ovarian cancer: final overall survival results from the PAOLA-1/ENGOT-ov25 trial	Ray-Coquard I. et al. (36)	2023

## 2 Characteristics of the HRD tests and their current recommendations

Identifying the BRCAness/HRD phenotype is clinically important to optimize the benefit of PARP inhibitors. Somatic or germline mutations of BRCA 1 and BRCA2 genes are the most frequent determinants of HRD profile, with a percentage of 15% for germline mutations and 6% for somatic mutations (5). However, this profile can also derive from somatic or germline mutations or methylations of other genes related to the HR mechanism (7). It is a complex scenario also related to dynamic mutational status, since it can change over time and in the natural history of the tumor. There may be indeed the restoration of an HRP status in the tumor cells through different mechanisms which lead to the consequent development of resistance to therapy with PARP inhibitors. For example there may be reversion mutations of the BRCA1/2 gene or RAD51C, RAD51D and PALB2, due to secondary genetic mutations, sometimes caused by long exposure to platinum-based chemotherapy and to the therapy with PARP inhibitors itself (37).

On the basis of the knowledge acquired on the genetics of ovarian cancer, the so-called HRD tests have been developed with the aim of identifying those tumors which, beyond the mutation of the BRCA1-2 genes, express a deficit of HR and against which a target therapy with PARP-inhibitors can be implemented. These tests can be classified into three categories (38). “Homologous Recombination Repair gene level tests” identify the etiology of HRD status, hence identify single somatic or germline mutations and methylations of the promoters of all the genes involved in HR mechanism through gene sequencing. “Genomic scars and signature tests” are capable of identifying tumors that express an HRD status by measuring tumor’s genomic scars and genomic instability, which are the result of somatic mutations accumulated over time (base substitutions, deletions and duplications that define loss of heterozygosity, allelic imbalance and other type of genomic instability phenomenon). Lastly, “Functional assays” provide a description of the real time, actual HRD or HRP status of the tumor and are currently being defined and evaluated in preclinical studies.

The myChoice CDx and the FoundationOne CDx test represent the companion tumor tests used to assess HRD status in the main pivotal studies regarding PARP-inhibitors and they are part of the second category of tests listed above. These are therefore tests that combine the search for BRCA1-2 mutations with a genomic instability score, which is obtained from the combination of different mutations and genetic variants identified using Next Generation Sequencing techniques on tumor tissue; this score describes the genomic scar of the tumor and therefore identifies tumors with a history of HRD rather than the current HR status of the tumor.

The FoundationOneCDx test (39) characterizes the HRD status by quantifying the loss of heterozygosity (LOH) or the presence of insertions and deletions, copy number alterations, gene rearrangements, etc. that frequently occur in HRD cells, since they use the Non Homologous End Joining mechanism to repair damage to the DNA double helix, a non-error-free mechanism like HR. This test also provides information about a tumor mutational burden and microsatellite instability. In clinical practice, the cut- off used to define a LOH high (hence HRD) or LOH low (hence HRP) tumor is 16%. Myriad’s myChoice CDx test (40) provides a genomic instability score (GIS) by combining the levels of heterozygosity loss, telomeric allelic instability and large-scale state transitions (LST) shown in DNA extracted from tumor tissue specimen. High HRD scores (GIS - high) are associated with very unstable tumors such as those with BRCA mutation, while low HRD scores (GIS – low) indicate genetically stable tumors. The cut off used to define a GIS high or GIS low tumor is 42.

Following the ESMO consensus of 2020 (38), the execution of the BRCA test is recommended to identify patients who derive most benefit from PARP inhibitors and should receive that therapy; in the context of the maintenance setting after first line chemotherapy, it is also recommended to perform the HRD test to establish the extent of the benefit from PARP-inhibitors in BRCA wild type and platinum sensitive patients - knowing that the LOH high/GIS high patient subgroup responds well to PARP inhibitor treatment. In the post-relapse maintenance therapy setting, BRCA and HRD testing can be performed to predict the magnitude of benefit from PARP therapy in the context of the benefit/risk assessment of that therapy.

## 3 Limitations of current HRD testing and development of functional assays

An important consideration is that currently available tests are not sensitive to changes in HRD status and do not provide a dynamic result, but a result that describes the genomics of the tumor at the time of tumor tissue sampling. Ideally, in recurrences, HRD testing should consequently be performed on a newly collected tissue sample and not on the previously archived tissue sample. In literature, several studies are evaluating the role of liquid biopsies, as an alternative to tissue biopsies, capable of collecting fragments of circulating tumor DNA, in order to identify those reversion mutations at the basis of chemo-resistance (17). Liquid biopsies may also be used in targeted proteomics to identify the over or under-expression of specific proteins, involved in key oncogenic signaling pathways associated to patient outcome, chemo-resistance and progression of disease. For example, disruption of the normal functioning of the PI3K/Akt/mTOR and MAPK pathway and overexpression of cyclin E were found to be related to resistance to platinum-based chemotherapy (26). Proteomics, such as mass spectrometry and protein array analysis, is a promising field of research. Firstly, it could identify any links between protein expression or alterations and tumor growth, representing a step forward to the development of a possible screening for ovarian cancer; secondly, it could predict chemotherapy response early identifying those adaptative mechanism of resistance in cancer cells, such as epigenetic changes and protein network rewiring through post-translational modifications. Lastly, it could ease the identification of targeted treatment against protein pathways involved in tumor progression and resistance to common chemotherapy (41). Targeted quantitative proteomics has some limitations, mostly due to its limited number of proteins that can be valued per analysis and to availability and quality of antibodies currently used, however it is definitely an emerging field that deserved to be developed and improved in order to find reliable proteomics biomarkers that can integrate genomics analysis.

On the other hand, there is a need to develop functional assay or tests capable of providing the genomic profile and therefore the HR status in a precise phase of the natural history of the tumor or in a precise moment in the therapeutic decision process, thus managing to identify even those cases in which the ability to activate HR has been recovered. The most promising functional assay under study is the quantification of RAD51 filaments (42); they are visualized by immunofluorescence techniques as spots, representative of the accumulation at the nuclear level of said protein, mediated by the BRCA1 and 2 proteins, when there is damage to the DNA double helix. The inability to form RAD51 complexes is therefore an expression of an HRD profile. Consequently, it is possible to calculate an HR score based on the number (or percentage) of RAD51 positive cells compared to the total number of cells in the S/G2 phase in order to functionally define the current HRP or HRD status of the cell, without necessarily identifying the specific cause. Therefore, it is necessary to standardize this assay as regards the adequacy of the tumor biopsy and the variability from observer to observer in counting the foci underlying the HRD score, which represent its current main limitations.

Finally, the studies performed with organoids are remarkably promising, i.e. culture models developed using tumor tissue taken from patients whose cells organize themselves anatomically and functionally in a similar way to the tumor, preserving their histological characteristics, heterogeneity, expression of biological markers and gene profile, as well as recreating the tumor microenvironment. They could in fact be used to perform functional HRD tests and to test a patient’s sensitivity to available therapies, as highlighted in a recent review (22).

## 4 Development of academic tests

At present, Myriad MyChoice CDx represents the gold standard as commercially available HRD test (38), however it has some limitations as a variable amount of samples can returned with inconclusive or false-negative results (43) and, as mentioned above, the identification of scars does not detect possible mechanisms of resistance and does not relate to the real time genomics of tumor. Moreover, Myriad MyChoice CDx test is only centrally performed, test availability and turnaround times differ from country to country depending on specific logistic policies and, mostly, it has high cost and, in Italy for example, it is not reimbursed by the National Healthcare System. For these reasons, the European Network of Gynecological Oncological trial groups (ENGOT) (23) has undertaken the “HRD initiative” in which PAOLA-1 trial’s tumor samples were shared with European academic centers in order to develop innovative, cheap, yet trusted academic alternative HRD tests.

The Italian team lead by Fumagalli et al. (27) suggests the AmoyDx HRD Focus Panel as an in house HRD-testing, which analyzes single nucleotide variants of BRCA1/2 and additionally gives a Genomic Scar Score (GSS) (44) by demonstrating it as a feasible alternative to Myriad Mychoice CDx test with an overall agreement between two test of 87.8% and a negative predictive value (NPV) of 100%. Excellent results are achieved by the Leuven HRD testing (45), which detects mutation in eight HR genes and analyzes about 90.000 genome wide single nucleotide polymorphisms using Next Generation Sequencing: it is reliable not only on detecting HRD tumors but also as a predictive value of efficacy of PARP-inhibitors. Lastly, an academic new approach to HRD testing is proposed based on MITO16A/MaNGO-OV2 trial population (28). In this study two genetic instability tests (LAB1 and LAB2) and a functional test (LAB3: RAD51 functionality) were compared with MyriadMychoiceCDx: LAB 1 and LAB2 showed a high concordance and a very low failure rate with the HRD status although a lower concordance for BRCA status with Myriad; LAB3 showed a failure rate of 30% (probably due to low quality paraffin-embedded samples) however, when discordant results occurred, RAD51 foci assay could detect additional HRD patients compared to Myriad. Failure of academic tests can depend on low quality samples due to small biopsies or poorly preserved paraffin-embedded samples which lead to suboptimal DNA quality. Nevertheless, academic tests are promising and there is increasing interest in their development.

## 5 PARP inhibitors

The introduction of PARP inhibitors has revolutionized the management of ovarian cancer both in maintenance therapy after first line chemotherapy and in the maintenance after rechallenge of platinum based-treatment. The mechanism of action of this class of drugs is based on their ability to bind, inactivate and catalyze PARP-1 and PARP-2, the most abundant nuclear PARP proteins at cellular level which, following single-strand damage of DNA, get bound to the double helix and recruit other proteins involved in the single strand break repair (SSBR) mechanism. The loss of function of PARP proteins causes an increase in mutations and an accumulation of DNA damage, which, being unrepaired, no longer involves the single strand only but also affects the double helix. Damage to the double helix could therefore be repaired through HR; however, if the cell (in this case a tumor cell) has a mutation of BRCA1/2 or other genes involved in DNA repair mechanism, the result is the cell cycle arrest, chromosomal instability and cell death. This process, through which PARP inhibitors cause the death of the tumor cell, is defined as “synthetic lethality “ (46). PARPi impair the ability to respond to DNA damage in cancer cells with BRCA-ness or HRD profile. However, this effect does not seem limited only to this class of patients, but it is extended to those who do not have mutations in known genes involved in HR, as demonstrated by the main studies on the subject.

If we consider that the first PARP inhibitor molecule was discovered more than 20 years ago (47), the development of PARP-inhibitor drugs and the evaluation of their efficacy in clinical trials is part of the recent scientific history.

The SOLO-1 study, conducted in 2018, and subsequent *post-hoc* analysis studies (34), evaluated the efficacy of 2-year maintenance therapy with Olaparib monotherapy in BRCA1/2 mutated patients and showed that, at a follow-up of 7 years, 67% of patients in Olaparib arm were alive (versus 46% of the placebo group) and 45% (versus 20%), respectively, had not received a first subsequent treatment. This randomized phase III study therefore demonstrated an improvement in OS and disease-free interval in BRCA-mutated patients treated with Olaparib as maintenance therapy. The PRIMA study (18) published in 2019 evaluated instead the efficacy of Niraparib as maintenance therapy after 1st line chemotherapy in patients with advanced ovarian cancer and high risk of recurrence (presence of residual tumor after debulking surgery, non-operable stage III, stage IV, patients who received neoadjuvant chemotherapy), stratifying patients according to Recombination mutational status and also including the group of Overall Population. The study demonstrated the benefit of maintenance therapy with Niraparib in terms of PFS in the HRD group of patients (maximum efficacy in the BRCAmut/HRD group with a PFS of 22.1 months versus 10.9 months in the placebo group, in any case with significant improvement also in the BRCAwt/HRD group with a PFS of 19.6 months versus 8.2 months in the placebo group). More importantly, this trial demonstrated an improvement in PFS also in the HRD-negative or HRP group (8.1 months versus 5.4 months) and in the Overall Population (13.8 months versus 8.2 months). These results led to the approval of Niraparib as maintenance therapy in all patients, regardless of mutational status, after response to first-line platinum-based chemotherapy. The PAOLA-1 study published in 2019 (19) investigated the efficacy of adding Olaparib to maintenance therapy with Bevacizumab in patients with advanced ovarian cancer treated in first line with platinum–taxane and Bevacizumab and it demonstrated the efficacy, in terms of prolongation of PFS, of this combination in patients with HRD, particularly in BRCA mutated patients (at 24 months 76% of the patients had not relapsed compared to 39% of the patients in the control group). However, no effective improvement was recorded in the group of HRD-negative or with unknown mutational status (PFS of 16.9 months versus 16 months). It should be underlined that this study did not include an Olaparib-only arm, therefore, to date, it is difficult to know whether the improvement in PFS is due to a greater extent to the PARP-inhibitor or rather to the synergistic effect of the two drugs combined. Recently Ray-Coquard and coll (36). confirmed an improvement in OS (5-year OS of 65.5% versus 48.5%) of the Olaparib plus Bevacizumab arm respect of maintenance therapy in the HRD population. Additionally, an update of PFS data showed that, at 5-year follow-up, 46% of HRD patients in the Olaparib plus Bevacizumab arm were alive and had not relapsed compared with 19% of patients in the Bevacizumab-only arm.

The ATHENA-MONO study published in 2022 (29) evaluated the efficacy of Rucaparib as maintenance therapy after response to 1st line chemotherapy in a very large patient population (independent of HRD status or residual tumor after surgery), subsequently stratifying the results by subgroups based on HRD status; a statistically significant benefit emerged in all patients in the Rucaparib group, including the HRD negative ones and also those with residual tumor after surgery (therefore high-risk patients), compared to the placebo group.

Comparing the results of the above-mentioned studies and those present in the literature is very difficult as a matter of methodology and design of these studies. In fact, the populations examined are profoundly different: some studies consider a very large population that includes both patients at high risk of recurrence and at low risk (see ATHENA-MONO study), others consider only one of the two categories (see high risk in the PRIMA study and low risk in the SOLO-1 study). In this context, at the 2022 ASCO meeting data from the Chinese PRIME study were presented (48): the study evaluated the safety and efficacy of Niraparib by stratifying patients according to response to 1st line chemotherapy (complete response versus partial response) thus dividing the patients into two distinct risk classes showing a rather divergent median PFS compared to that obtained in the PRIMA study, in which only high-risk patients were enrolled (24.8 months vs 13.8 months). Moreover, a greater prolongation of PFS was registered in the group with complete response to chemotherapy (29.4 months) compared to the group of patients with partial response to chemotherapy (19.3 months), indicating response to 1st line chemotherapy as a possible predictor of response to PARP inhibitor therapy. Another study that divided patients according to risk classes is that of Harter et al. (30), a *post-hoc* analysis of PAOLA-1. In this study, the efficacy of the combination of Bevacizumab and Olaparib was evaluated not only on the basis of HRD status as in the PAOLA-1 study, but by further dividing the patients into two risk classes: high risk, represented by stage III patients undergoing upfront surgery with residual tumor or undergoing neoadjuvant chemotherapy and patients in stage IV; and low risk, represented by patients in stage III undergoing upfront surgery with no residual tumor. Regarding residual tumor and surgical management, recent studies (13, 49) underline how the activity of PARP-inhibitors is similar in patients with and without residual disease after surgery, however patients with complete gross resection followed by PARP-inhibitors therapy show greatest improvement in PFS rates. This implies the need to achieve complete cytoreduction as a surgical target in patients with ovarian cancer.

Finally, another methodological point that makes difficult the comparison of studies is the use of different tests to determine the HRD status: BRCAnalysis in the SOLO-1, PRIMA and PAOLA-1 study was conducted using Myriad myChoice, while in ATHENA-MONO, ARIEL and NOVA study was adopted FoundationOne.

Literature proposes several meta-analyses which evaluate the efficacy of PARP inhibitors both as maintenance therapy after first line chemotherapy (8) as maintenance therapy in platinum-sensitive recurrence (10) confirming the recommendation for the use of PARP inhibitors in patients with ovarian cancer, albeit with different levels of benefit based on the HRD phenotype. It therefore appears evident that BRCA and HRD status substantially change the therapeutic strategies in the treatment of ovarian cancer. The current challenge is to investigate the potentiality of combine PARPi with other therapeutic agents in order to enhance efficacy and avoid resistance, for example anti-angiogenic agents, i.e. Bevacizumab, and immunotherapy, i.e. anti-programmed cell death 1 (PD-1) antibodies, anti-programmed cell death ligand-1 (PD-L1) antibodies and anti-human cytotoxic T-lymphocytic-associated (CTLA4) antibodies. In this regard, immune checkpoint inhibitors may achieve great results. Despite the presence of an highly immunosuppressive tumor microenvironment that causes poor recognition of tumor cells by the immune system, it is shown that BRCA mutated and HRD ovarian cancers express higher levels of neoantigens because of defect in DNA repair mechanisms (31). Moreover, PARP inhibitors are suggested to upregulate PD-L1 expression and stimulate interferon-mediated immune response having therefore a synergic action in immune stimulation. An overview of future expectations and ongoing trials is reported by Vanacker et al. (50) showing a wide development of this field of research.

## 6 PARP-inhibitors and their current recommendations

In Italy, the 2021 Associazione Italiana Oncologia Medica (AIOM) guidelines (51) recommend the following: the use of Olaparib as maintenance therapy in patients with high-grade serous and endometrioid cancer, stage III-IV, with BRCA mutation, in partial or complete response after platinum-based chemotherapy; the use of Niraparib is indicated as maintenance therapy in patients in partial or complete response after platinum-based chemotherapy with stage III and residual disease after surgery or stage IV, regardless of mutational status. In platinum-sensitive recurrence, after rechallenge with platinum-based chemotherapy, the AIOM guidelines also suggest maintenance therapy, based on Bevacizumab or PARP-inhibitors, depending on which maintenance therapy has been proposed after first line. In this setting, Olaparib is suitable for BRCA mutated patients, Niraparib and Rucaparib for all comers (albeit with greater benefits in BRCA mutated or HRD patients).

NCCN’s American guidelines of 2021 (12) are similar to the Italian and European ones, although it is important to consider the presence of different approval policies regarding the methods and settings for the use of PARP inhibitors in Europe and in America (52). Olaparib has been approved by both the Food and Drug Administration (FDA) and the European Medicines Agency (EMA) as maintenance therapy in BRCA-mutated patients after platinum-based first line chemotherapy and in HRD positive patients after first line chemotherapy based on platinum and Bevacizumab and has been approved as maintenance therapy in relapse regardless of mutational status. Niraparib is approved by both the FDA and EMA as maintenance therapy after first-line platinum-based chemotherapy, both in partial response and in complete response, regardless of mutational status. Rucaparib is approved by both FDA and EMA as maintenance therapy in platinum-sensitive relapse after platinum-based chemotherapy; in patients undergoing two or more lines of chemotherapy, FDA has approved Rucaparib as monotherapy in BRCA-mutated patients, while with an information note dated 21 July 2022, EMA withdrew its approval for this indication after analyzing the results on Overall Survival (53).

It should be underlined that reimbursement policies differ from country to country. In Italy, for example, Olaparib is reimbursed when used, after partial or complete response to first line chemotherapy, only in patients in HRD/mutated patients, whereas Niraparib is reimbursed only as a maintenance therapy after primary debulking surgery with residual tumor in stage FIGO III or interval debulking surgery and a partial or complete response to first line chemotherapy or in stage IV patients with ovarian cancer regardless of mutational status.

## 7 Pending issues

### 7.1 Negative test patients

It seems increasingly necessary to talk about patients with negative result at HRD test rather than truly HRD-negative patients. The tests currently used for pivotal studies and clinical trials (Myriad Mychoice CDx or FoundationOne CDx) may in fact fail to identify patients with other mutations which manage to determine a Homologous Recombination deficit. From a recent consensus of experts regarding the BRCA/HRD test, the need to develop new, alternative and validated tests for the identification of genomic instability and mutational status of HR emerged for clinical practice and the choice of therapy after first-line chemotherapy (32).

### 7.2 PARP-inhibitor response prediction models

To date, the main predictor of a good response to PARP inhibitor therapy is sensitivity to platinum therapy (11, 20). BRCA 1-2 mutations, both somatic and germline, are also predictive of a positive response to PARPs inhibitor (38), yet taking into account the possibility of secondary mutations or reversions affecting these genes, capable of restoring, even partially, the ability of the cell to use HR and which cannot be identified with HRD tests currently on the market.

Somatic mutations in non-BRCA homologous recombination genes confer an advantage in terms of PFS in platinum treatment, when compared with patients who have neither BRCA mutation nor HRD (54). HRD status and LOH assessment therefore have a prognostic value. As to their use as predictive markers, the results are still conflicting: the ARIEL2 study (55), which evaluated the efficacy of Rucaparib in patients with platinum-sensitive recurrence of ovarian cancer, showed that BRCA mutated patients and BRCAwild-type but LOH high patients provide a significant response to Rucaparib compared to BRCA wild type but LOH low patients. The study suggests that assessment of LOH status could be used as a predictive marker of sensitivity to PARP inhibitors in platinum sensitive and BRCAwt patients, broadening the pool of patients who could benefit from these drugs. LOH status as a predictive marker is currently applicable only to relapsed patients treated with one or two lines of chemotherapy but not to patients undergoing 3 or more lines of chemotherapy (24) and has not yet been validated in maintenance settings after first line chemotherapy in first diagnosis. A point that must further be taken into account is that the cumulative predictive value of the mutations of the genes involved in HR, determining the level of LOH, is considered: it could be relevant to understand if sensitivity to PARP-inhibitors is different according to the single mutations and how each mutation contributes to determining such sensitivity.

Another predictive factor highlighted in the ARIEL2 study is the methylation level of the BRCA gene promoter: in particular, high methylation levels, biologically related to reduced expression of the genes of which they are promoters, seem to be associated with good sensitivity to PARP inhibitors. This result is consistent with that conducted by Kondrashova et al. in 2018 (16): using xenograft models (patient derived xenograft) or original tumor tissue transplanted into immunodeficient guinea pigs, the study demonstrated how the state of zygosity of the BRCA promoter methylation changes the response to PARP inhibitors (the drug implied in this case was Rucaparib), highlighting how the homozygous - hence of both alleles – methylation of BRCA was predictive of a good response to PARP-inhibitors, unlike heterozygous methylation which was, on the contrary, associated with resistance to therapy. Furthermore, comparing the methylation status of chemo naive tumor tissue biopsies and those after treatment, this study showed how the methylation status can change after chemotherapy treatment due to the effect of platinum on DNA, in which there can be a transition from homozygosity to heterozygosity and vice versa, with subsequent development of resistance or sensitivity to PARP inhibitors. As mentioned previously, HRD status is dynamic, which has two important implications: on the one hand, the result of available HRD tests is, at present, inconsistent, even if promising, as an absolute predictive marker of response to therapy and, on the other hand, there is a need for an immediate introduction of PARP-inhibitor therapy in sensitive patients to obtain the maximum benefit from this class of drugs.

Another study that used xenotransplantation and investigated the possibility of a preclinical model of response to PARP inhibitors is Chen et al’s dated 2022 (56) which suggests the gene expression levels of KRAS and ATK1 and the levels of the CA125 marker < 10 mIU/ml after chemotherapy as possible new predictive biomarkers of response to PARP inhibitors, also in BRCAwt, HRD negative and platinum resistant patients.

### 7.3 BRCA-mutated patients not responding to PARP-inhibitors

40% of BRCA mutated patients with ovarian cancer do not respond to PARP inhibitors after first line chemotherapy agents (21, 57). This may be due to the presence of reversals of the BRCA gene mutation, the development of replication fork protection mechanisms, loss of function of p53 Binding Protein 1 (53BP1) or reduced expression of the PARP-1 protein (20, 21). In this sense, the quantification of RAD51 strands has shown promising results in the ability to identify cases of treatment resistance due to reduced expression of 53BP1 or BRCA reversion mutations, in identifying BRCA mutated tumors that nonetheless prove HRP depending on RAD51 foci expression and finally in its use as a predictive marker of response to platinum and PARP inhibitors (42). With regard to the latter, the study by Guffanti et al. (33) evaluated, using patient-derived ovarian cancer xenografts, the baseline expression levels of RAD51 foci and demonstrated how the percentage of RAD51 positive cells correlates inversely with the response to Olaparib and directly with the risk of recurrence and platinum resistance. The study results are consistent with those obtained for breast cancer (58).

Moreover, it is known that BRCA2 mutation has a better prognosis than BRCA1 mutation, as it is associated with a better response to platinum-based chemotherapy and with greater survival chances (30, 48) in consideration of the different biological function of the two proteins. However, a recent meta-analysis (25) demonstrated that this prognostic difference is not evident in the context of the response to PARP-inhibitors, the benefit of which is comparable between the two mutations. Finally, it should be underlined that there is growing evidence in literature of how BRCA 1-2 mutations are not the same in all cases, but there is the possibility that the site and type of mutation of the BRCA1-2 genes actually correlates with a different response to PARP inhibitors and with possible development of chemoresistance (15, 59, 60). A *post hoc* analysis in the subgroup of BRCA mutated patients of PAOLA-1 trial (35) was recently conducted to evaluate the predictive value of the location of BRCA1-2 mutations in terms of efficacy of PARP- inhibitors maintenance. Interestingly, patients with mutation in the DNA binding domain (DBD) of BRCA2 had lower rate of relapse, suggesting a great platinum-sensitivity, whereas patients with mutation in the DBD of BRCA1 were at high risk of relapse with a lower PFS, showing less platinum-sensitivity and similar survival to that of non-carriers but, unexpectedly, were extremely sensitive to Olaparib. Moreover, the magnitude of benefit of PARP-inhibitors was found to be different depending on location of BRCA mutations, in particular women with a BRCA1 mutation in the RING domain respond worse to PARP-inhibitors therapy than those with mutations in the DBD. Domain-related sensitivity to platinum and PARP-inhibitors represents a new and promising field of study and need to be deeply investigated in order to develop an even more tailored, genetically and biologically, therapy for treat ovarian cancer.

## 8 Conclusion

Knowing that more than 70% of patients diagnosed with HGSOC recur within 3-5 years, clinical research has recently focused on identifying the best maintenance therapy to improve survival.

Combining the clinical need of maintenance and knowledge on the genomics of ovarian cancer, PARP- inhibitors have been developed and they have had the most important impact on the history of ovarian treatment over the last 5 years. PARP inhibitors exhibit higher efficacy in BRCA mutated patients, but they are also effective, though to a lesser degree, in HRD-positive patients, HR proficient patients and patients with unknown mutational status. BRCA mutations and HRD status are therefore important factors in ovarian cancer diagnosis and treatment decisions. The detection of these genomic features influences treatment options and patient outcomes in terms of progression free survival and overall survival. HRD testing is recommended by the main European and American guidelines and there is an urgent need to have reliable tests in order to broaden the number of patients that can benefit from PARP-i therapy. There still remain some issues that need to be solved related to limitations of current HRD genomic test used in pivotal studies. On the one hand, they can’t identify all HRD positive patients, as shown by the fact that even some HRP patients respond to PARP-i. For this reason, it would be more correct to refer to patient with negative test as HRD testing doesn’t reliably exclude all patients who don’t benefit from PARP-i maintenance. Moreover, such tests are not sensitive to dynamic changes in tumor genetics, which throw reversion or secondary mutations of HRR genes, they can restore an HR proficiency status and cause chemo-resistance. Therefore, functional assays are under development, providing a real time genomic status. They will improve our ability to select appropriate patients who benefit from PARPi. Equally promising are clinical studies featuring organoids and xenograft models in order to perform functional HRD tests and to test a patient’s sensitivity to available therapies. Creation of models that can predict chemotherapy and PARP-i sensitivity and response, by integrating clinical, histological and molecular data, are under study.

In conclusion, HR status has a prognostic and predictive value. Improvements in the knowledge of genes involved in the HR pathway and the clinical significance of their mutations are making it possible to personalize cancer treatment. Molecular characterization of cancer is changing the clinical oncological approach and, in the future, the development of more accurate and precise genomic tests may allow to chose the best therapy for each patient and change the history of ovarian cancer patients.

## Author contributions

AM: Data curation, Formal analysis, Writing – original draft, Conceptualization, Validation. VT: Data curation, Writing – original draft. CA: Supervision, Validation, Writing – review & editing. SR: Conceptualization, Supervision, Writing – review & editing. AB: Visualization, Writing – original draft. EP: Visualization, Writing – original draft. GD: Supervision, Visualization, Writing – original draft. AE: Supervision, Writing – original draft. LD: Supervision, Validation, Visualization, Writing – original draft. AF: Supervision, Visualization, Writing – original draft. DL: Conceptualization, Validation, Writing – original draft. GS: Supervision, Validation, Writing – original draft. GV: Supervision, Validation, Visualization, Writing – original draft.
